# An Efficient and Stable Polarizing Agent for In-Cell Magic-Angle Spinning Dynamic Nuclear Polarization NMR Spectroscopy

**DOI:** 10.1021/acs.jpclett.4c02709

**Published:** 2024-11-11

**Authors:** Yu Rao, Pierrick Berruyer, Andrea Bertarello, Amrit Venkatesh, Marinella Mazzanti, Lyndon Emsley

**Affiliations:** † Laboratory of Magnetic Resonance, Institut des Sciences et Ingénierie Chimiques, École Polytechnique Fédérale de Lausanne (EPFL), 1015 Lausanne, Switzerland; ‡ Group of Coordination Chemistry, Institut des Sciences et Ingénierie Chimiques, École Polytechnique Fédérale de Lausanne (EPFL), 1015 Lausanne, Switzerland

## Abstract

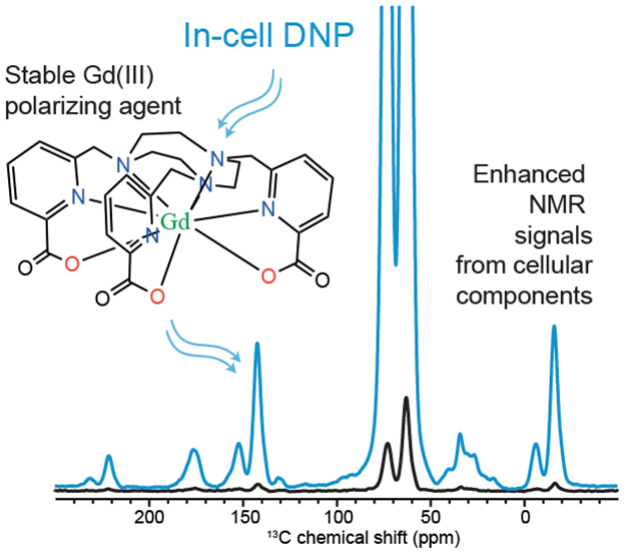

Nuclear Magnetic Resonance (NMR) spectroscopy would be a method of choice to follow biochemical events in cells because it can analyze molecules in complex environments. However, the intrinsically low sensitivity of NMR makes in-cell measurements challenging. Dynamic Nuclear Polarization (DNP) has emerged as a method to circumvent this limitation, but most polarizing agents developed for DNP are unstable in reducing cellular environments. Here, we introduce the use of Gd(III)-based DNP polarizing agents for in-cell NMR spectroscopy. Specifically, we show their persistent stability in cellular formulations, and we investigate the DNP performance of the Gd(III)-based complex [Gd(tpatcn)] in human embryonic kidney cell lysates and intact cells. For cell lysates, DNP enhancements up to −27 are obtained on the cellular signals, reproducible even after storage at room temperature for days. Mixing the [Gd(tpatcn)] solution with intact cells enables the observation of cellular signals with DNP, and DNP enhancement factors of about −40 are achieved.

Following biochemical events in viable human cells is a key objective in chemical biology. Indeed, cells are highly complex environments which can only be reproduced partially in model materials. Nonetheless, biophysical methods for in-cell investigation at the molecular level are still limited.

Nuclear Magnetic Resonance (NMR) would be a method of choice because of its ability to analyze unmodified molecules even in complex environments. Liquid-state NMR has been successfully used for a range of in-cell characterizations.^1−10^ These include for example protein structure determination in human cells,^4,11^ monitoring metabolic activity in cells^12^ and characterizing lipid dynamics in membranes,^13^ or following post-translation modifications.^14^ Unfortunately, NMR is hampered by its intrinsically low sensitivity, making many in-cell measurements with NMR challenging. In the above examples, the sensitivity issue was circumvented by either overexpressing the protein of interest in the cells or by directly injecting a large quantity of isotopically enriched molecules of interest, leading to analyte concentrations that are far from physiological.

To address the sensitivity problem in NMR, hyperpolarization methods have been developed to break the limit of thermal equilibrium polarization. Notably, Dynamic Nuclear Polarization (DNP) enhances NMR signals via polarization transfer from unpaired electron spins to nearby nuclear spins, and can enable up to a 2 orders of magnitude increase in transient nuclear polarization. In particular DNP is now well established for use in magic angle spinning (MAS) solid-state NMR spectroscopy,^15^ to study a wide range of substrates, from surface structures,^16^ and battery materials,^17^ to zeolites,^18^ pharmaceuticals^19−21^ and biomolecules.^22^

MAS DNP has also been successfully applied to cellular samples to enhance the NMR sensitivity of frozen, lysed and intact cells,^23−27^ for example to characterize specific protein interactions^28−32^ or to quantitatively determine drug concentrations at micromolar level in cells.^33^

DNP NMR experiments require unpaired electron spins as a source of polarization. In most samples, as unpaired electrons are not naturally present, they are most simply introduced by impregnating the sample with a solution containing a stable radical.^34,35^ In MAS DNP NMR the sample is then frozen, at around 100 K to lengthen electron spin relaxation times, thus enabling efficient DNP.^36^

Current state-of-the-art in-cell DNP NMR uses nitroxide-based organic stable radicals, such as AMUPOL,^37^ as the DNP polarizing agent (PA).^38^ However, nitroxide radicals are not stable in reducing environments as they can easily be transformed to the DNP-inactive hydroxylamine form.^39^ Unfortunately, the cellular environment is reductive and nitroxide-based DNP agents are quickly transformed to the DNP-inactive form.^40^ For this reason, the use of nitroxide radicals for in-cell DNP faces stability issues and usually requires short incubation times to limit radical reduction.^23,24^ Tuning the substituents around the nitroxide radical center was found to slow down this reduction process, but also reduced the DNP enhancement, and does not fully prevent reduction.^41,42^

On the other hand, another class of DNP polarizing agents based on Gd(III) chelate complexes^43−46^ possess excellent redox stability: Gd(III) cannot be reduced in water. Gd (III) complexes have previously been used to perform DNP experiments in the presence of reducing agents such as ascorbic acid.^44^ The reported value of the reduction potential of Gd(III) to Gd(0) is −2.29 V and that of the complex [Gd(tpatcn)] is expected to be even lower.^47^ Indeed, Gd(III) complexes have been used as contrast agents for MRI in clinical applications, and the reduction of the Gd(III) center is never a concern for in vivo imaging.^48^ Different to the strategies of slowing reduction by protecting nitroxide radicals, Gd(III) is thermodynamically stable which in principle leads to an infinite lifetime in the cellular environment.

Here, we introduce the use of Gd(III)-based DNP polarizing agents for in-cell NMR spectroscopy. Specifically, we investigate the DNP performance of the Gd(III)-based complex [Gd(tpatcn)] in HEK cell lysates and in intact HEK cells. As previously shown by Stevanato et al., [Gd(tpatcn)] is, so far, the best performing Gd(III) complex for DNP^44^ (where tpatcn is 1,4,7-tris[(6-carboxypyridin-2-yl)methyl]-1,4,7-triazacyclononane). We find that [Gd(tpatcn)] has long-term stability in cellular environments. For cell lysates, we find that DNP enhancements on the cellular components do not decay after several days, demonstrating the stability of the PA in this reductive environment. When suspending intact HEK cells in a glycerol–water solution of [Gd(tpatcn)], we observe DNP enhancements of more than a factor 40 for the cellular components.

The dominant DNP mechanism for Gd(III) complexes is the solid effect (SE).^46^ It relies on saturation of the forbidden double-quantum or zero-quantum transitions of the electron–nucleus two spin system. It was found earlier that the EPR line width of the central transition is dominated by the zero-field splitting (ZFS), with the DNP performance of Gd(III) polarizing agents as a result being dominated by the symmetry of the coordination environment of the Gd(III) center.^44,45^ So far, [Gd(tpatcn)] has been found to provide the highest ^1^H DNP enhancements.^44^ As mentioned above, Gd(III) complexes exhibit very high redox stability in aqueous solution. Thus, the oxidation state of Gd(III) complexes, when used in physiological or biological conditions, such as in contrast agents for MRI, is extremely stable. Notably, Stevanato et al. showed that [Gd(tpatcn)] can hyperpolarize ascorbic acid, which typically reduces and rapidly deactivates most nitroxide-based radicals.^44^ Both the Gd(III) center and the chelating ligand are found to be inert in this reducing environment.

To assess the stability of [Gd(tpatcn)] in the cellular environment, we prepared solutions of the complex in HEK293T cell lysates. Figure 1a-c shows X-band electron paramagnetic resonance (EPR) EPR spectra recorded before the addition of cell lysate (Figure 1a) and 180 and 360 min after the addition of the cell lysate (Figure 1b and c). The double integration of the EPR spectra allowed us to derive the concentration of the [Gd(tpatcn)] in the solution as a function of time, which is shown in Figure 1d. This test was also performed with TEMPO, the typical building block of binitroxide DNP radicals, and with AMUPol,^37,49^ the most commonly used DNP polarizing agent for in-cell DNP, and the results are shown in Figure 1e-h and 1i-l, respectively. The TEMPO signal decayed rapidly upon mixing with the cell lysate due to the reduction of the radical by the reducing chemicals in the cells, with a half-life of under 30 min. The complete reduction of AMUPol in cell lysate is overall slightly slower than TEMPO (with a half-life for the total radical concentration of ∼60 min), but the pattern in the EPR spectrum undergoes a dramatic change. The spectrum changes very significantly from t = 0, which corresponds to the spectrum of AMUPol, to t = 60 min since the reduction of AMUPol occurs in two-steps, and where at t = 60 min there is now a significant population of monoradicals in which only one side of the AMUPol has been reduced. This species is paramagnetic, but is a much less efficient polarizing agent.^25,41^ This is in line with previous studies of the reduction of AMUPol in cellular environments.^27^ In contrast, Figure 1d shows that the concentration of [Gd(tpatcn)] is constant in the cell lysates throughout the whole 6 h duration of the measurement, corresponding to no reduction of [Gd(tpatcn)] on this time scale. Equally importantly, Figure 1 shows that the EPR peak width remains unchanged, indicating that the coordination environment of the Gd(III) center was not affected by the molecules in the cell lysate.^50^ Therefore, not only the Gd(III) center, but also the whole complex, is stable in the cell lysate. Additionally, we note that in the original publication introducing [Gd(tpatcn)] as a DNP polarization agent,^44^ [Gd(tpatcn)] was shown to yield a ^1^H enhancement of −37 for *d*_*8*_-glycerol/D_2_O/H_2_O 6:3:1_v/v_ with 1.5 M urea, which has a pH of 7. They also showed a ^1^H enhancement of −24 for 1.3 M ascorbic acid, which has a pH of 2–3. Thus, the robustness of [Gd(tpatcn)] enhancements across the range of pH present in cells has been established. Taken together, these results suggest that the DNP performance of [Gd(tpatcn)] should be preserved in cellular environments.

**Figure 1:**
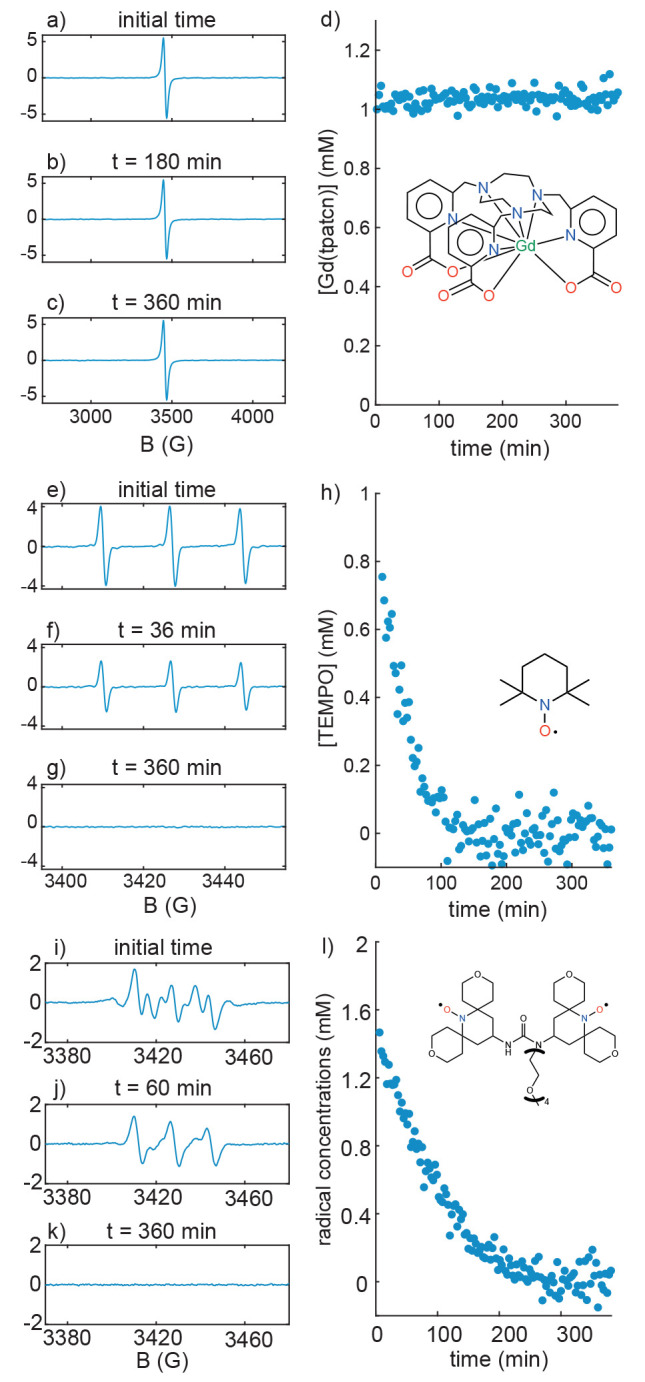
(a-c) Room temperature X-band solution-state EPR spectra of ∼1 mM [Gd(tpatcn)] at different times after mixing with HEK293T cell lysate ((a) initial mixing, (b) 180 min, (c) 360 min). (d) [Gd(tpatcn)] concentration in the solution as a function of time. (e-g) Room temperature X-band solution-state EPR spectra of ∼1 mM TEMPO at different times after mixing with HEK293T cell lysate ((e) initial mixing, (f) 36 min, (g) 360 min). (h) TEMPO concentration in the solution as a function of time. (i-k) Room temperature X-band solution-state EPR spectra of ∼1 mM AMUPol at different times after mixing with HEK293T cell lysate ((i) initial mixing, (j) 60 min, (k) 360 min). (l) AMUPol concentration (labeled as overall radical concentration) in the solution as a function of time. The concentrations are calculated from the EPR spectra.

To evaluate the performance of [Gd(tpatcn)] as a DNP polarizing agent in such conditions we first performed 9.4 T MAS DNP experiments with the cell lysates. The magnetic field was finely tuned to match the maximum negative DNP enhancement of [Gd(tpatcn)].^44^ Lysate samples were prepared as described above. The samples were then frozen and measured at ∼100 K at a MAS rate of 8 kHz.

The resulting ^1^H–^13^C cross-polarization (CP) MAS spectra are shown in Figure 2. The signal at 175 ppm arises from the carbonyl carbons of proteins.^51^ The peak at 30–35 ppm is assigned to the aliphatic carbon CH_2_ signals of the lipidic components of the cells.^52^ The peak at ∼0 ppm comes from the silicone plug used for sealing the rotor, and the two strong peaks at 63 and 73 ppm are from glycerol. Comparing the spectra with (green line) and without (black line) microwave (μwave) irradiation, all the peaks except for the silicone plug are negatively enhanced. For convenience, the spectrum with μwaves is shown after multiplication by a factor of −1. The DNP enhancement of the solvent is a factor −27.2 ± 0.2. A reference solution of 4 mM [Gd(tpatcn)] in *d*_*8*_-glycerol/D_2_O/H_2_O 60:30:10_v/v_ gave an DNP enhancement of −48.1 ± 0.2. The DNP enhancement of the solvent is reduced with respect to the reference solution most probably because of the more abundant protons in the lysate, as the hydrogen in the cells is in natural abundance.^53^ As for the signals from the cellular components, the C=O peak and the aliphatic peak are enhanced by factors of −27 ± 5 and −23 ± 3, respectively, which are both similar to the solvent enhancement. Indeed, the complex containing lysate solution is quite homogeneous, and we expect that the DNP enhancements are identical among the different components of a homogeneous mixture.^53,54^

**Figure 2:**
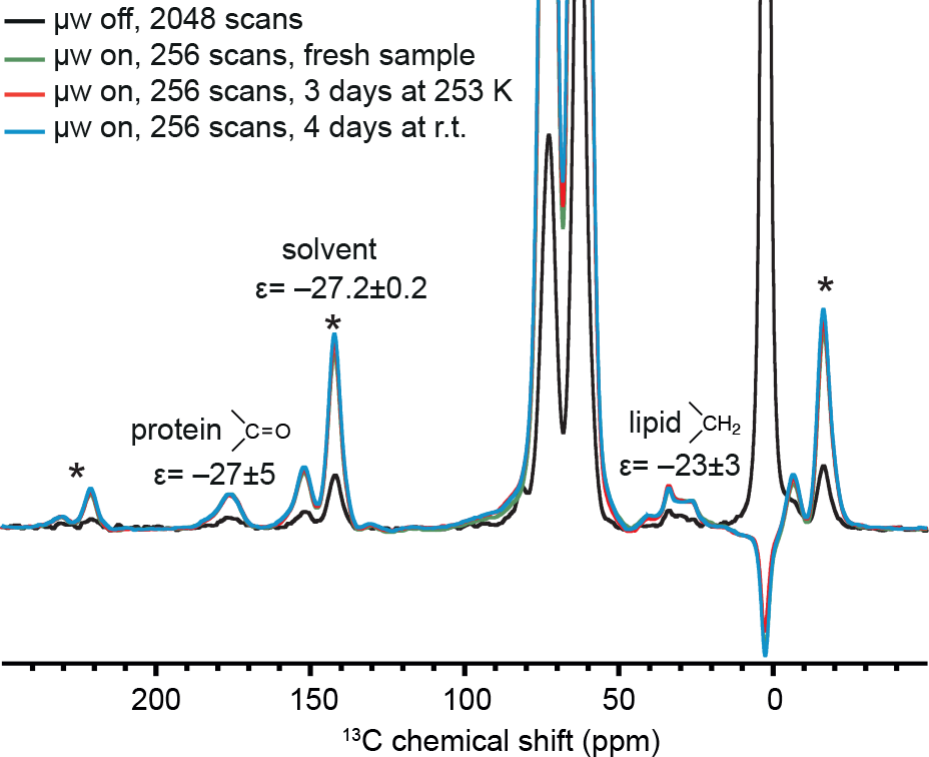
^1^H–^13^C CPMAS spectra of lysates of 3 million HEK293T cells in 20 μL of a 4 mM solution of [Gd(tpatcn)] in *d*_*8*_-glycerol/D_2_O/H_2_O 60:30:10_v/v_ with (green/red/blue) and without (black) microwave irradiation. The measured DNP enhancements are labeled for each peak. The microwave on spectra were collected on a fresh sample (green) and after the sample was kept at 253 K for 3 days (red) and then at room temperature for 4 days (blue). Note that the green, red and blue spectra are essentially identical. The spectra were acquired at 8 kHz MAS and ∼100 K on a 9.4 T NMR spectrometer. Spinning sidebands are marked with asterisks.

To examine the stability of [Gd(tpatcn)] in cell lysate over a longer time scale, Figure 2 also shows DNP enhanced spectra recorded after the sample was stored at −20 °C for 3 days (red), and then at room temperature for another subsequent 4 days (blue). (The corresponding microwave off spectra can be found in Figure S4). We observed that the microwave on spectra are identical. In addition, the DNP build-up times were measured and are reported in Figure S5 and Table S2, and they were also found to be consistent with the initial values. These results demonstrate that the [Gd(tpatcn)] complex exhibits exceptional stability within cell lysates, even during storage at room temperature.

We then examined the stability and performance of [Gd(tpatcn)] for intact HEK cells. As described above, 3 million HEK293T cells were suspended in 15 μL of the [Gd(tpatcn)] solution in *d*_*8*_-glycerol/D_2_O/H_2_O 60:30:10_v/v_. The sample was packed into a sapphire rotor and closed with a zirconia drive cap. The rotor was then immersed in liquid nitrogen and freezes in a few seconds. Then the frozen sample was quickly transferred to the precooled DNP MAS probe. The transfer method described by Frederick et al.^24^ was used to prevent sample melting which would lead to partial lysing of the cells. As shown in ref^33^ and by the dye exclusion test shown in Figure S10, the cells lose their cell membrane integrity, but mostly maintain their intact shape under this treatment, i.e. they do not lyse. Using this sample preparation protocol, we prepared a set of samples varying the concentration of [Gd(tpatcn)] from 4 to 16 mM. Table 1 reports the DNP enhancements measured with these different samples. The highest cellular material enhancements were obtained using an 8 mM solution of [Gd(tpatcn)] in *d*_*8*_-glycerol/D_2_O/H_2_O 60:30:10_v/v_. Figure 3 shows the CP spectra of that sample acquired with and without μwave irradiation. The spectra for the other two concentrations are shown in SI (Figures S6, S7). For the solvent peak, an enhancement of –44.4 ± 0.3 was obtained, which is similar to the enhancement of −48.1 ± 0.2 measured on a bulk reference solution of 8 mM [Gd(tpatcn)] in *d*_*8*_-glycerol/D_2_O/H_2_O 60:30:10_v/v_. Note that both the cellular components (carbonyl peaks and the aliphatic peaks) are enhanced by factors of nearly −40. This difference between the solvent enhancement and that of the cellular materials is observed in all the samples reported in Table 1, where the enhancements of the cellular components are slightly lower than the solvent enhancement (except for the carbonyl peak as measured at 16 mM [Gd(tpatcn)], but this value is subject to a large error). The large error range may contribute to the observed differences in enhancements between the C=O and CH_2_ resonances at this concentration, but this could also be due to differences in local proton concentrations.

**Figure 3:**
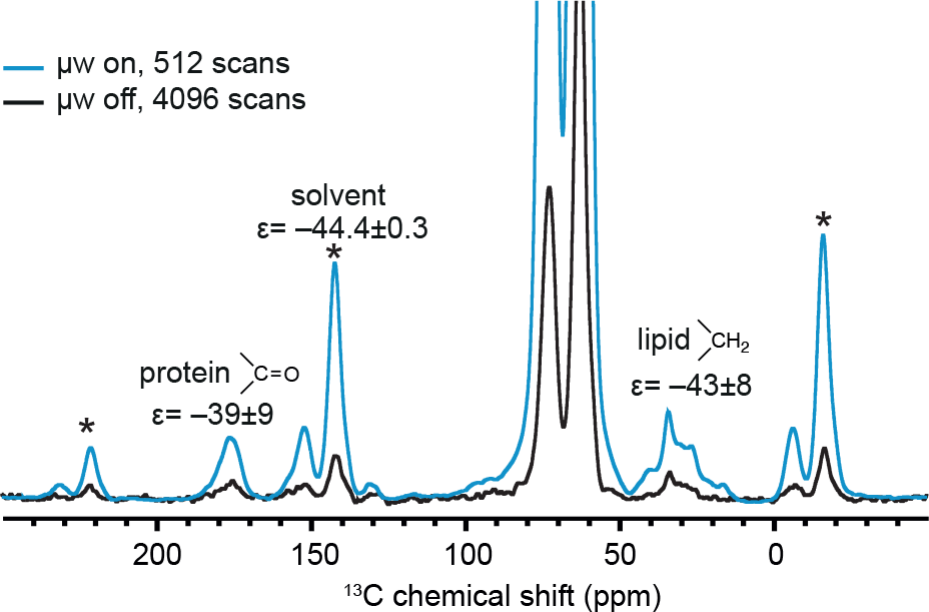
^1^H–^13^C CPMAS spectra of 3 million HEK293T cells suspended in 15 μL of an 8 mM solution of [Gd(tpatcn)] in *d*_*8*_-glycerol/D_2_O/H_2_O 60:30:10_v/v_ with (blue) and without (black) microwave irradiation. The measured DNP enhancements are shown for each ^13^C peak. The spectra were acquired at a MAS rate of 8 kHz and ∼100 K on a 9.4 T spectrometer (400 MHz for ^1^H).

**Table 1:** DNP enhancements obtained with different concentrations of [Gd(tpatcn)]

[Gd(tpatcn)] concentration (mM)	Number of HEK293T cells	ε(solvent)	ε(C=O)	ε(CH_2_)	*T*_b_^on^ (s)
4	3M	–35.9 ± 0.2	–21 ± 4	–23 ± 3	19.7
8	3M	–44.4 ± 0.3	–39 ± 9	–43 ± 8	8.0
16	3M	–30.1 ± 0.2	–38 ± 14	–21 ± 3	3.0
4	0	–48.1 ± 0.2			11.5

In this context, we can hypothesize that [Gd(tpatcn)] can either diffuse or not through the cell membrane. If [Gd(tpatcn)] does not diffuse through the membrane, then the whole cell would have to be polarized by ^1^H spin diffusion. ^1^H spin diffusion spontaneously transports polarization from hyperpolarized regions to nonhyperpolarized areas. Pinon et al.^54^ have shown that, depending on the sample, the polarization can travel across distances from nm to μm within the sample. A clear signature of this process is the difference of the DNP enhancement between the region where the complex is located and the region hyperpolarized via ^1^H spin-diffusion. However, here, the size of a typical HEK293 cell is on the order of 10 μm. With the experimental conditions here (notably the observed nuclear T_1_ (μwave off)), we estimate the spin diffusion length (*D*_*H*_ = 330 *nm*^2^*s*^–1^) to be <0.1 μm.^53^ The difference by 2 orders of magnitude between the cell size and the spin diffusion length clearly rules out the possibility that the cells are only hyperpolarized by relay from the bulk solvent by spin diffusion.

The remaining explanation for the observed enhancements is that the complex can diffuse through the cell membrane, allowing the generation of local hyperpolarization in the cells that will depend on the local concentration of [Gd(tpatcn)] and on the local proton density. As discussed above, in line with previous observations,^24^ the cells are not viable after being treated with high concentrations of glycerol, and as a consequence the cell membrane no longer has selective permeability. Under these conditions, [Gd(tpatcn)] is expected to penetrate into the cells after treatment and before freezing and generate local hyperpolarization.^24,55^

In this respect, Stevanato et al. showed that the optimal concentration of [Gd(tpatcn)] in frozen solutions is 4 mM, yielding a DNP build-up time of T_b,on_ = 12 s.^44^ As shown in Table 1, if the [Gd(tpatcn)] concentration of the solution used to suspend the cells is 4 mM, we measure T_b,on_ of the solvent signal to be 19.7 s. This value is longer than the reference value, and is closer to the value measured for a [Gd(tpatcn)] concentration of 2 mM (17.8 s).^44^ This is consistent with the idea that the DNP polarizing agent crosses the cell envelope, thus reducing the [Gd(tpatcn)] concentration in the extracellular environment. Consistent with this, in cell suspensions in 8 mM and 16 mM solutions of [Gd(tpatcn)], the T_b,on_ are similar to the reported value for bulk [Gd(tpatcn)] solutions with lower concentrations of 6 mM (T_b,on_ = 8.7 s) and 12 mM (T_b,on_ = 3.3 s), respectively.^44^

While the use of 60% *d*_*8*_-glycerol in the DNP solvent is expected to prevent cell lysing upon freezing,^24,33^ maintaining cell viability (in the sense that they are positive for the Trypan Blue test) requires a more advanced protocol that has been developed by Fredericks and co-workers.^24^ Their protocol relies on the use of *d*_*8*_-glycerol/D_2_O/H_2_O 15:75:10 to suspend the cells, followed by slow freezing of the sample from room temperature to–80 °C at a rate of 1 °C/min.^24^ Following this protocol, we performed DNP using [Gd(tpatcn)] to hyperpolarize viable HEK293T cells, and the resulting spectra are shown in Figure 4. In this case, two phases with different polarizing agent concentrations were observed, as the signals build up with two components of comparable size but with different times (7 and 300 s, as shown in Figure S9). This separation is caused by the low-glycerol medium, but is not related to the slow freezing since it is also observed in a flash-frozen solution of 4 mM [Gd(tpatcn)] in the same medium (Figure S3). Consequently, the DNP is hampered since the solvent signal is only enhanced by a factor of −7.9 ± 0.5, (which is consistent with the frozen solution without any cells (ε = −7 ± 1)) and is significantly lower than in 60% *d*_*8*_-glycerol, as shown in Table 1. As shown in Figure 4, the enhancements of the two cellular signals are −3.2 ± 0.5 and −2.7 ± 0.3, which are lower than the solvent enhancement. Despite the overall low enhancement, the difference between the enhancements of the solvent and the cell resonances is much larger than for the intact but nonviable cells used in Figure 3, in line with the expectation that penetration of [Gd(tpatcn)] into the cells is greatly reduced when the cells are viable. We note that the loss in overall enhancement when using a low-glycerol formulation is not specific to the use of [Gd(tpatcn)], and that biphasic behavior and proportionally lower enhancements are also observed when using nitroxide radicals.^24,32^

**Figure 4:**
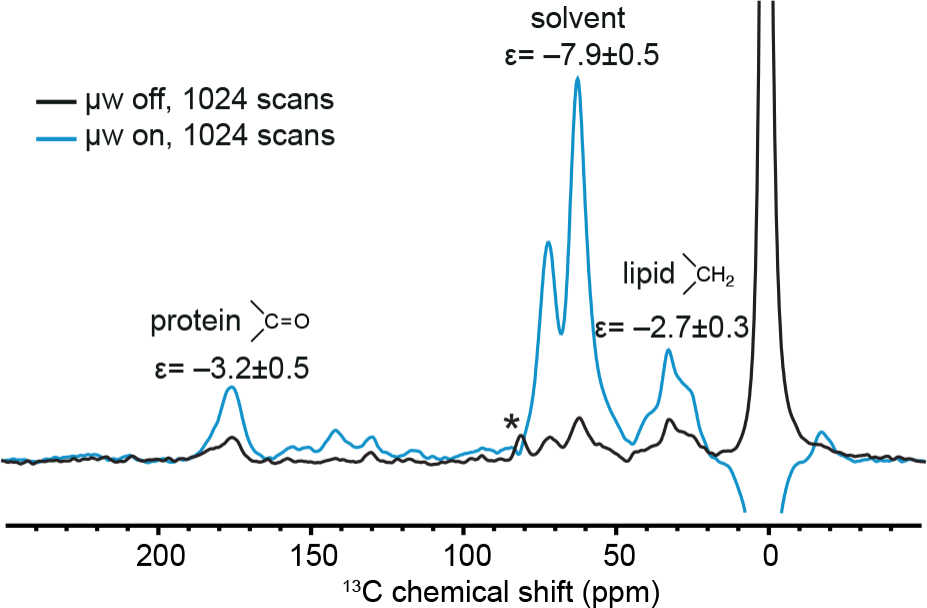
^1^H–^13^C CPMAS spectra of 3 million HEK293T cells suspended in 15 μL of a 4 mM solution of [Gd(tpatcn)] in *d*_8_-glycerol/D_2_O/H_2_O 15:75:10_v/v_ with (blue) and without (black) microwave irradiation. The DNP enhancements are labeled for the different peaks. The spectra were acquired at 8 kHz MAS and ∼100 K on a 9.4 T spectrometer (400 MHz for ^1^H).

In conclusion, we have shown that [Gd(tpatcn)] can be used as a polarizing agent for in-cell DNP enhanced NMR. Due to the redox stability, [Gd(tpatcn)] overcomes the reduction issues associated with using nitroxide-based radicals as polarizing agents. For the cell lysate, a DNP enhancement of −27 was obtained on the cellular signals, and which is reproducible even after storage at room temperature for days. Mixing the [Gd(tpatcn)] solution with intact cells enables the direct observation of signals from cells with DNP, and DNP enhancement factors of about −40 were achieved by tuning the bulk [Gd(tpatcn)] concentration. The results described here will allow more efficient application of in-cell MAS DNP NMR.

## Supporting Information Available

Experimental details, additional NMR spectra, and a link to all the raw NMR data (PDF)
