# Supplementary material for: An Efficient and Stable Polarizing Agent for In-Cell Magic-Angle Spinning Dynamic Nuclear Polarization NMR Spectroscopy
Source: J Phys Chem Lett. 2024 Nov 11;15(46):11601–7. doi: 10.1021/acs.jpclett.4c02709 (PMC11587084; doi:10.1021/acs.jpclett.4c02709)
Supplement: Supplementary file 1 — jz4c02709_si_001.pdf [file jz4c02709_si_001.pdf]

Supporting Information for:

An Efficient and Stable Polarizing Agent for In-Cell Magic Angle Spinning Dynamic Nuclear Polarization

Yu Rao,[a] *Pierrick Berruyer,* [a],† *Andrea Bertarello,* [a],+ *Amrit Venkatesh,* [a],$ *Marinella Mazzanti,* [b] *Lyndon Emsley*[a],*

[a]Institut des Sciences et Ingénierie Chimiques, Ecole Polytechnique Fédérale de Lausanne (EPFL), CH-1015 Lausanne, Switzerland

[b]Group of Coordination Chemistry, Institut des Sciences et Ingénierie Chimiques, École Polytechnique Fédérale de Lausanne (EPFL), 1015 Lausanne, Switzerland

Corresponding author:

*lyndon.emsley@epfl.ch

Present Addresses

†School of Chemistry and Biochemistry, University of Geneva, 1211 Genève, Switzerland.

$Dr. Amrit Venkatesh: National High Magnetic Field Laboratory, Florida State University, 1800 East Paul Dirac Drive, Tallahassee, Florida 32310, United States.

Table of Contents

**Supplementary Information**	**Page**
Raw data statement	S3
S1.1 Materials and Sample Preparations	S4
S1.2 NMR Experimental	S5
S2.1 Reference DNP experiments	S6
S2.2 Cell lysate DNP experiments	S8
S2.3 Cell DNP experiments	S10
S2.4 Cell DNP experiments in 15% glycerol	S12
S3 Trypan blue test	S13
References	S14

Raw data statement

The NMR raw data is available (http://doi.org/10.5281/zenodo.14050675) in the original TopSpin, JCAMP formats. Data are made available under the license CC-BY-4.0 (http://creativecommons.org/licenses/by/4.0/ Creative Commons Attribution 4.0 International).

S1.1 Materials and Sample Preparations

Materials

[Gd(tpatcn)] was prepared according to the reported protocol.[1] The PBS buffer was purchased from Gibco and the other chemicals such as TEMPO and d_8-glycerol were obtained from Sigma-Aldrich. HEK293T cells were provided as suspensions in Lysogeny broth (LB) by the EPFL protein production and structure core facility.

Sample preparation for EPR

3 million HEK293T cells were pelleted at 200*g* in an ultracentrifuge for 5 min to remove the supernatant. After washing twice with 100 µL of PBS buffer, the cell pellet was suspended in 20 µL of a 2 mM solution of [Gd(tpatcn)], TEMPO, and AMUPOL in D_2O and then lysed by 4 freeze (liquid-nitrogen)-thaw cycles. Finally, the mixture was loaded into a capillary for X band CW EPR measurement on a Bruker X-band EMX Nano spectrometer. The measurements were performed continuously over six hours to monitor the change in the signals.

Sample preparation of lysed cells for DNP NMR

For the cell lysate, 3 million HEK293T cells were pelleted and washed as described above. Then the cells were lysed in 20 µL of 4 mM solutions of [Gd(tpatcn)] in d_8-glycerol/D_2O/H_2O 60:30:10 by 8 freeze-thaw cycles. The solution was transferred to a 3.2 mm sapphire rotor, sealed with a silicone plug and closed with a zirconia cap.

Sample preparation of intact cells for DNP NMR

For the intact cells, 3 million HEK293T cells were pelleted and washed as described above. Then the cells were suspended in 15 µL of 4, 8 or 16 mM solutions of [Gd(tpatcn)] in d_8-glycerol/D_2O/H_2O 60:30:10. After being transferred to 3.2 mm sapphire rotors, and closed with a zirconia cap, the whole rotors were quickly frozen in liquid nitrogen and then inserted into the precooled NMR probe. Note that no silicone plugs were used for intact cell samples because the samples are pre-frozen and thus not subject to sample leaks.

For the DNP experiments on viable cells using 4 mM [Gd(tpatcn)] in d_8-glycerol/D_2O/H_2O 15:75:10, a slow freezing process of 1 ºC/min from room temperature to –80ºC was applied using a Corning CoolCell LX Cell freezing container.

S1.2 NMR Experimental

DNP-enhanced MAS NMR experiments were performed on a 9.4 T (400 MHz for ^{1}H) Bruker DNP NMR spectrometer with a gyrotron as the microwave source, a low temperature cooling system, and a 3.2 mm triple resonance low temperature MAS DNP probe operating at ca. 100 K. The gyrotron was operating with approximately 25 W of output power measured at the probe base. The magnetic field was finely tuned using a Lakeshore power supply unit (Model 625) to match the solid-effect DNP condition for ^{1}H. All DNP experiments were performed at a sample spinning frequency of 8 kHz. Standard pulse sequences for ^{1}H-^{13}C cross polarization (CP) and ^{1}H direct acquisition were used. ^{1}H build-up times were measured using a saturation recovery pulse sequence with spin echo detection. The recycle delay of the experiments was set to 1.3×T_b. The ^{1}H-^{13}C CP spectra were referenced by calibrating the most intense peak of glycerol to 63.0 ppm. Key NMR parameters are provided in Table S1.

Table S1. Typical experimental parameters used in MAS DNP experiments.

**^{1}H direct**	
π/2 pulse	2.5 μs
Acquisition time	8 ms
Recycle delay	15 s
**^{1}H-^{13}C CP**	
^{1}H π/2 pulse	2.5 μs
CP contact time	3 ms
^{13}C CP power level	60 kHz rf
^{1}H CP power level	40 kHz rf
Acquisition time	20 ms
Recycle delay	15 s
SPINAL-64 ^{1}H decoupling	100 kHz rf

S2.1 Reference DNP experiments

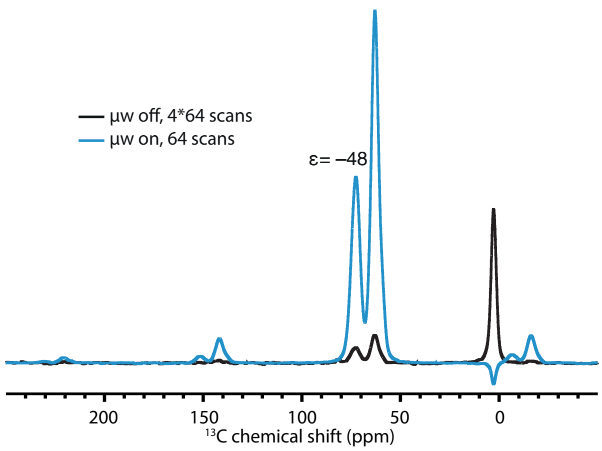

Figure S1. ^1H-^{13}C CP spectra of 4 mM Gd(tpatcn) in d_8-glycerol: D_2O: H_2O = 60: 30: 10 with (blue) and without (black) microwave irradiation at the field position where maximum DNP enhancement was obtained. As the DNP enhancement is negative, a 180° of 0th-order phase correction was applied to the spectrum with microwave irradiation. The spectra were collected by a recycle delay of 15 seconds and the microwave off spectrum has the number of scans four times of the microwave on spectrum.

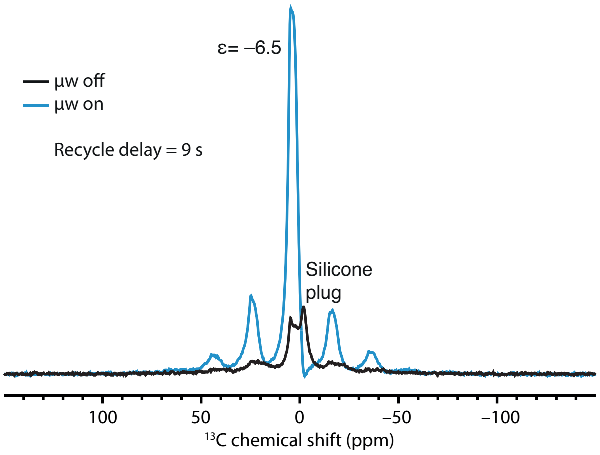

Figure S2. ^1H spectra with (blue) and without (black) microwave irradiation of 4 mM Gd(tpatcn) in d_8-glycerol: D_2O: H_2O = 15: 75: 10 at the field position where maximum DNP enhancements were obtained. As the DNP enhancements were negative, a 180° of 0th-order phase correction was applied to the spectrum with microwave irradiation. The spectra were collected by a recycle delay of 9 seconds.

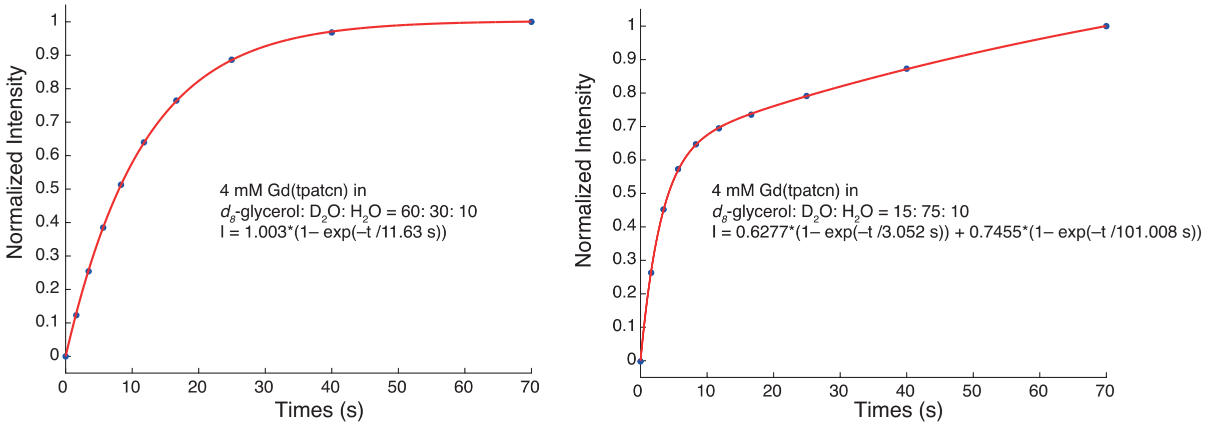

Figure S3. Plots showing the fits of the experimental T_b^{on}, DNP build-up times of the two reference DNP experiments in Figure S1 and S2, under microwave irradiation.

S2.2 Cell lysate DNP experiments

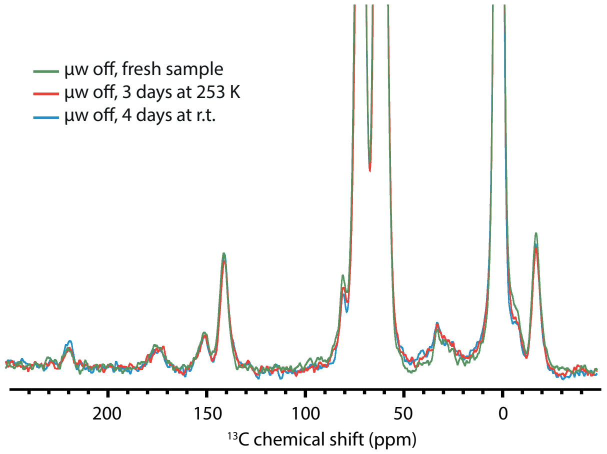

Figure S4. ^{1}H-^{13}C CP spectra of 3 million HEK293T cells lysate in 20 μl of 4 mM Gd(tpatcn) d_8-glycerol/D_2O/H_2O 60:30:10$_{v/v}$ solution without microwave irradiation (green). They were also collected after the sample was kept at 253 K for 3 days (red) and at room temperature for 4 days (blue). The spectra were acquired at 8 kHz MAS and about 100 K on a 9.4 T spectrometer (400 MHz for ^{1}H).

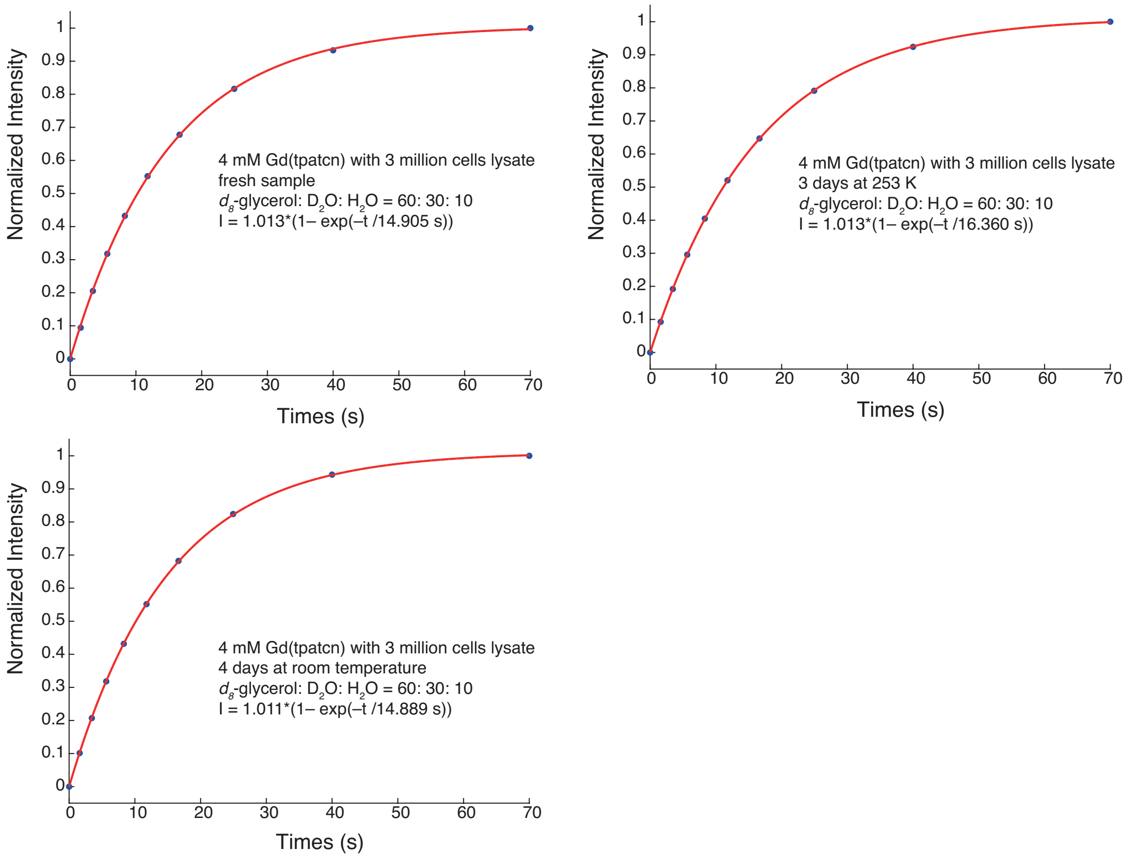

Figure S5. Plots showing the fits of the experimental T_b^{on}, DNP build-up times of the cell lysate DNP experiments in Figure 2, under microwave irradiation.

Table S2. DNP build up time and the recycle delays used in the DNP cell lysate experiments, the spectra in Figure 2.

Sample	T_b^{on} (s)	Recycle Delay (s)
fresh	14.9	20
3 days at 253 K	16.4	20
4 days at r.t.	14.9	20

S2.3 Cell DNP Experiments

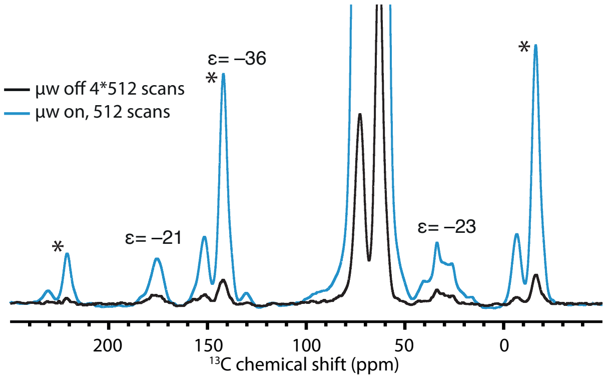

Figure S6. ^1H-^{13}C CP spectra of 3 million HEK293T cells suspended in 15 μl of 4 mM Gd(tpatcn *d*$_8$-glycerol/D_2O/H_2O 60:30:10$_{v/v}$ solution without (black) and with (blue) microwave irradiation and the DNP enhancements were labelled at the corresponding peak. The spectra were acquired at 8 kHz MAS and about 100 K on a 9.4 T spectrometer (400 MHz for ^1H).

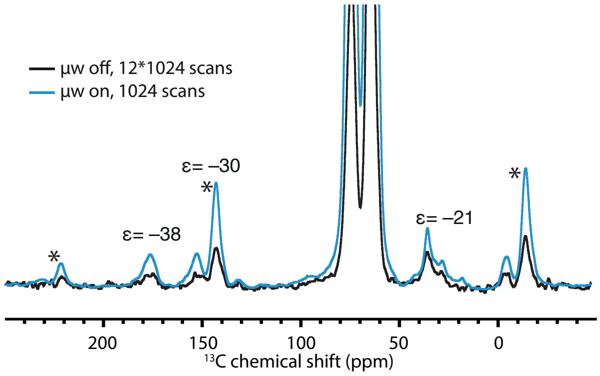

Figure S7. ^1H-^{13}C CP spectra of 3 million HEK293T cells suspended in 15 μl of 16 mM Gd(tpatcn d$_8$-glycerol/D_2O/H_2O 60:30:10$_{v/v}$ solution without (black) and with (blue) microwave irradiation and the DNP enhancements were labelled at the corresponding peak. The spectra were acquired at 8 kHz MAS and about 100 K on a 9.4 T spectrometer (400 MHz for ^1H).

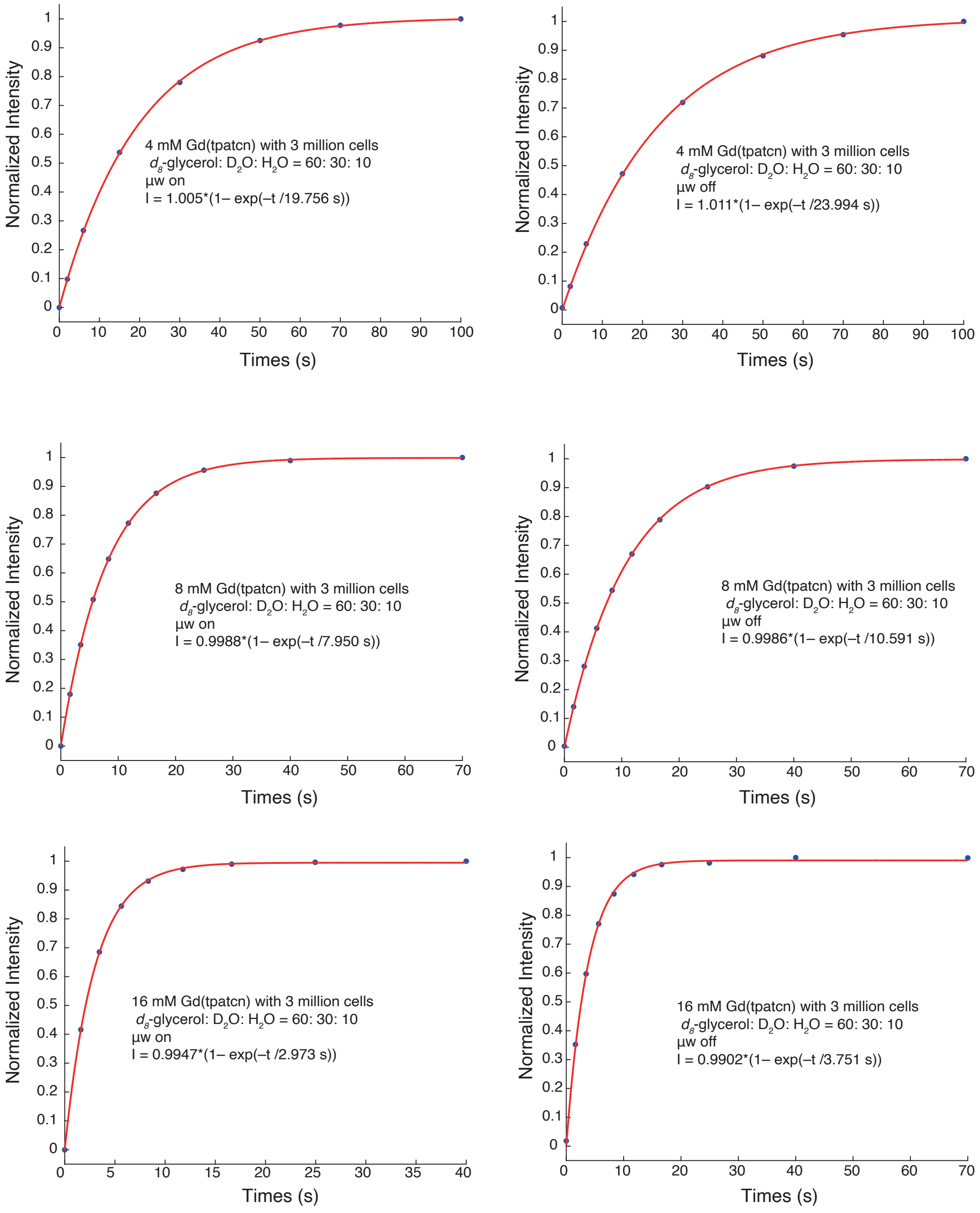

Figure S8. Plots showing the fits of the experimental T_b^{on} and T_1, DNP build-up times of the cell DNP experiments in Figure 3 and Figure S6 and S7, with and without microwave irradiation.

S2.4 Cell DNP experiments in 15% glycerol

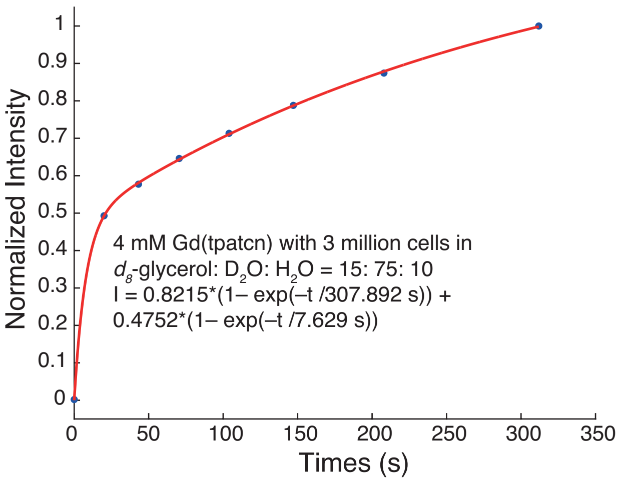

Figure S9. Plots showing the fits of the experimental T_b^{on}, DNP build-up times of the cell DNP experiments in Figure 4, under microwave irradiation.

S3 Trypan blue test

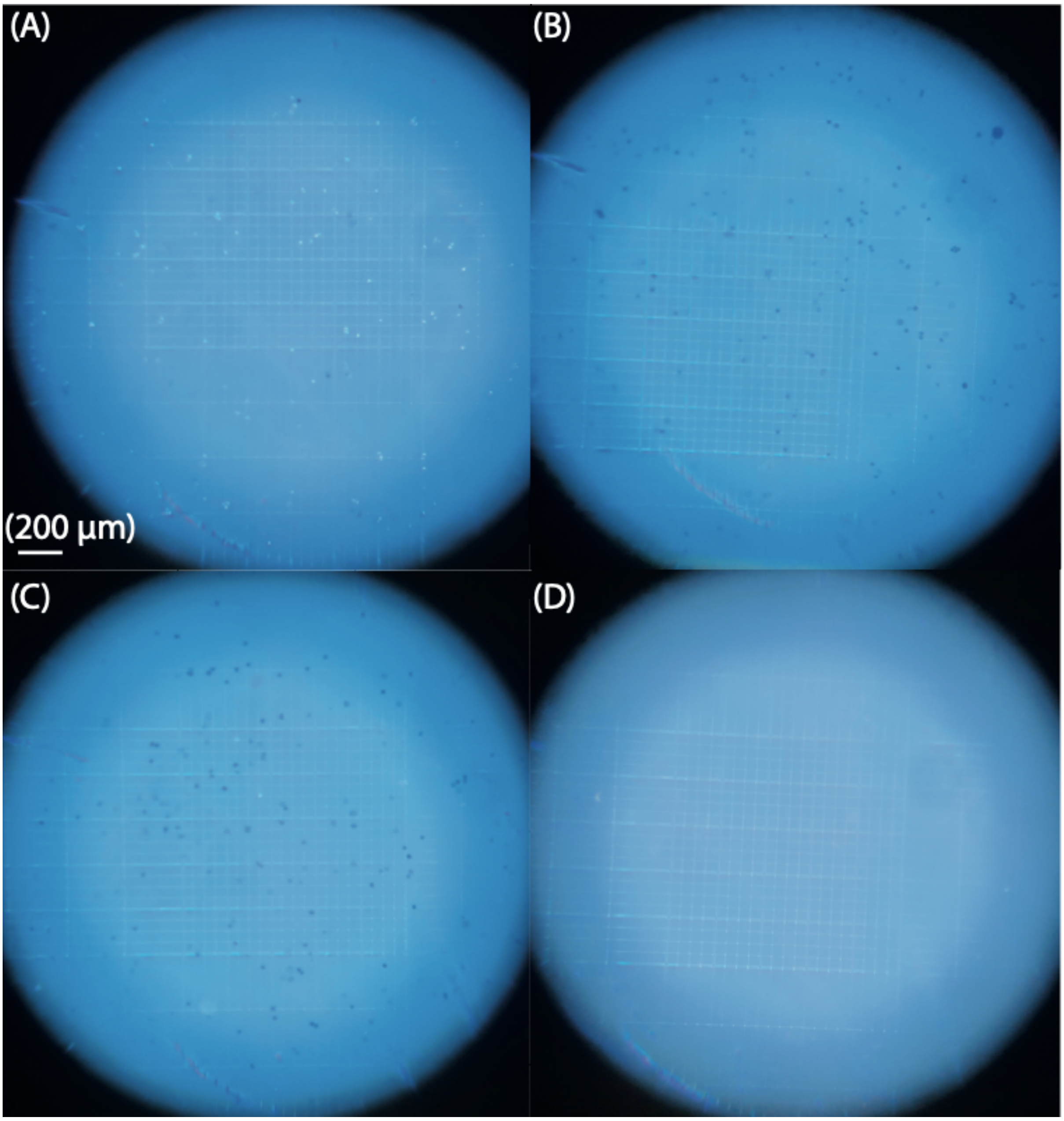

Figure S10. Trypan blue exclusion assays of HEK293T cells and divided in four identical aliquots (as described in the experimental). In this assay cells that are viable exclude the dye and appear as white dots and cells that remain intact but are not viable take up the dye and appear as blue dots. (A) Cells pelleted and resuspended in PBS buffer as a reference, show mostly viable. (B) Cells pelleted and resuspended in d_8-glycerol: D_2O: H_2O = 60: 30: 10, show mostly dead but intact cells. (C) Cells pelleted and resuspended in 4 mM of Gd(tpatcn) in d_8-glycerol: D_2O: H_2O = 60: 30: 10, show similar results with (B). (D) Blank reference without any cells. The small squares have

an edge length of 50 µm.

Reference

1. Gateau, C.; Mazzanti, M.; Pécaut, J.; Dunand, F. A.; Helm, L., Solid-state and solution properties of the lanthanide complexes of a new tripodal ligand derived from 1,4,7-triazacyclononane. *Dalton Trans.* **2003**, 2428-2433.
